# Making research articles fit for purpose: structured reporting of key methods and findings

**DOI:** 10.1186/s13063-015-0575-7

**Published:** 2015-02-20

**Authors:** Douglas G Altman

**Affiliations:** Centre for Statistics in Medicine, Nuffield Department of Orthopaedics, Rheumatology & Musculoskeletal Sciences, University of Oxford, Botnar Research Centre, Windmill Road, Oxford, OX3 7LD UK

‘If physicians are to base treatment decisions on the evidence in the medical literature, all the relevant results of trials must be available easily and consistently. Yet it is common to have trouble identifying the hypothesis, the research question, and the design of a published trial. It is even more common to lose count of the participants or to be unable to tell who received what therapies and the type of analysis used. As a result, it is often impossible to know whether the conclusions are justified by the data’ [1].

There is a big problem with journal articles. Readers of published research reports, especially systematic reviewers, struggle to find key details of study methods and often cannot extract the results they seek [1]. Many research articles are clearly unfit for purpose [2].

What is the solution? One way is to develop text-mining software to locate the relevant information, and several groups are working on such initiatives [3-5]. But text-mining is only a potential solution for extracting information that is in fact there, albeit hard to locate; it cannot assist at all for information that is simply absent from the research report. There is a wealth of evidence that key information is commonly missing from published reports of trials [6,7]. So while text-mining software could be useful for existing literature, going forwards with it is a solution to the wrong problem. Instead, I believe we need to consider a different format for publishing research results.

Research reports published in journal articles serve multiple readerships, each with different needs. Regardless of length and style, it is essential that such articles include all relevant details of the study methods and the key findings. Two fundamental principles are that a research article should include enough information about the methodology to allow others to replicate the study, and the results should be given in enough detail to enable them to be included in a subsequent systematic review and meta-analysis [8]. In principle the format of a report is irrelevant if those criteria are met. However, the current standard story-telling format embeds factual information and numerical results within the narrative text, making some details hard to extract and, crucially, masking the absence of essential material. These problems affect all types of research but the seriousness is arguably greatest in relation to reports of randomised trials, which I will consider here.

## Deficiencies in journal articles

Reporting guidelines emerged in the 1990s in response to the abundant evidence of missing or ambiguous information in published research reports [9,10]. The Consolidated Standards of Reporting Trials (CONSORT) Statement, first published in 1996 and most recently updated in 2010 [11], outlines the minimum information that should be included in reports of the results of randomised trials. Approaching 20 years since its first appearance, its benefit on reporting has been clearly seen, but with only modest improvement over time [12].

Several hundred journals have stated support for, or adoption of, CONSORT, but adherence remains inadequate. Dozens of recent reviews have shown that reporting of essential information continues to be generally inadequate in trial reports across all areas of medicine [6,7,13,14]. The present situation is clearly unacceptable. Various ideas are being explored to improve the quality of publications including some targeted at editorial processes. Indeed improved reporting has been demonstrated when editorial resources are focused specifically on adherence to CONSORT [15-17]. One possibility that needs serious consideration is to change the format of research articles by introducing more structure.

Structured reporting has been introduced in areas of clinical practice. Improved completeness and clinical value have been demonstrated, for example in pathology [18], radiology [19], and telemedicine [20]. Structure in research reports began with the adoption of the IMRAD format (Introduction, Methods, Results and Discussion) augmented by the adoption of structured abstracts [21]. Although in each case there were fears that the structure would stifle creativity, both succeeded because of their self-evident benefit on the quality of published articles [22].

## Structured reporting of research articles

Within medical research, trial registers provide a perfect example of structured reporting. So, for example, registering a study on clinicaltrials.gov requires completion of a structured template. Likewise, in the section for subsequent reporting of the results, researchers have to provide the study findings in a structured way. It has been shown that some results posted on clinicaltrials.gov, especially harms, are more complete than those in corresponding journal articles reporting the same trials [23,24].

Journal articles that report research findings are currently in a mainly narrative style, usually supported by tables and perhaps also figures. As Riveros and colleagues observed, ‘using templates with mandatory reporting of some elements may facilitate the work of researchers by reminding them what they need to report and by standardising their reporting’ [23]. It is timely to consider whether journal articles would be more informative if they had more structure.

There are two main forms of structure. First, more structure can be applied to the main text by creating many more sections. One of the antecedents of CONSORT was SORT (Standards of Reporting Trials), a reporting guideline that referred to structured reporting in its title [25]. The authors defined structured reporting as providing sufficiently detailed information about the design, conduct and analysis of the trial for the reader to have confidence that the report is an accurate reflection of what occurred during the various stages of the trial. A single published case study of using each item of the SORT checklist as a heading for a short section was widely considered to be a failure, as it made the article longer and less readable [22]. However, some of the criticisms were related more to the content of SORT than the format. Three subsequent generations of the development of CONSORT have addressed those concerns; it is not reasonable to continue to dismiss the principle of structure based on a single case study published 20 years ago using a different checklist. In fact, much the same idea has been implemented elsewhere. The journal *PLoS Clinical Trials*, published from 2006–2007, created a template based on the then current CONSORT checklist of 2001. Each item in the CONSORT checklist was used a heading in the article. Although this was in essence a repeat of the SORT approach, there were fewer headings and they were structured within the IMRAD format.

Alternatively, authors can identify in the text where each element of the CONSORT checklist is addressed [26]. This approach has no impact on the format of the article, but requires the authors to mark up the text. While that format should help to avoid omissions, some checklist items do not refer to a specific piece of information in a single location, and so this is not in my view a workable solution in general.

A different approach to structure is to adopt a more tabular format akin to completing a tax form, as is required for example on clinical trials registers. In fact, several structural elements are already routinely used in reports of randomised trials. Almost certainly the most successful impact of CONSORT has been the flow diagram, which is now included in the majority of published trial reports. The diagram depicts the flow of participants through the trial, from enrolment through allocation and follow up to analysis. Readers, including peer reviewers, can quickly see the numbers of randomised participants, identify when and why some were lost to follow-up, and the extent to which the numbers analysed reflect the numbers randomised. Other structures within most reports of trial results are a structured abstract and a table showing the baseline characteristics of each intervention group (usually as Table 1). But it is simple, and I believe desirable, to include other elements within explicit displays (tables or boxes), such as eligibility criteria, details of interventions, outcomes, and primary results. Currently, such information is rarely included as a display in published articles. The suggestion is intended to make key information easy to locate rather than to require a rigid format. Thus the same displays can easily accommodate the modified recommendations in several extensions to CONSORT (http://www.consort-statement.org/extensions). A similar idea has been proposed for articles reporting prognostic studies of tumour markers [27].

Readability is often put forward as an argument against structure. This would be a stronger argument if current research reports were highly readable, but many are not. One reason is that much factual information is embedded within the text, which works especially poorly for trial results. Readability is certainly desirable, but far more important are reliability and relevance [28], and crucially important too is reproducibility, which of course requires complete reporting. One reason for suggesting greater use of structural elements is to pull apart factual information from narrative, making it easier both to locate specific information and also to understand the broad sweep of a study without being diverted by all the details, important though they are.

Greater use of structure within research articles (not just for randomised controlled trials) would improve completeness by helping authors ensure they address key issues. It would also greatly aid reviewers and editors in appraising articles, and it would assist future systematic reviewers who currently struggle to find the key information they seek. Indeed, more structure would also aid text-mining. In the future, trial results will perhaps appear only in registries, presumably in a highly structured format, with journals carrying only narrative discussions of their findings [29]. Such a radical change does not seem imminent, however.

In the initial editorial published when *Trials* was launched, the editors wrote that ‘we believe that there is scope for new and better ways to report the findings of trials. *Trials* will develop and refine innovative approaches to improving communication about trials’ [30]. As yet there has been very little activity on this theme. We encourage contributions, both suggestions for suitable formats and also specific examples of real trial results presented in alternative formats. This editorial is thus the first contribution in a new *Trials* series on ‘New ways to publish research findings’.

## Abbreviations

CONSORT: Consolidated Standards of Reporting Trials
IMRAD: Introduction, Methods, Results and Discussion
SORT: Standards of Reporting Trials
